# Centralized colorectal cancer screening outreach and patient navigation for vulnerable populations in North Carolina: study protocol for the SCORE randomized controlled trial

**DOI:** 10.1186/s43058-021-00194-x

**Published:** 2021-10-07

**Authors:** Teri L. Malo, Sara Y. Correa, Alexis A. Moore, Renée M. Ferrari, Jennifer Leeman, Alison T. Brenner, Stephanie B. Wheeler, Xianming Tan, Daniel S. Reuland, on behalf of the Scaling Colorectal Cancer Screening Through Outreach, Referral, and Engagement (SCORE) Study Protocol Consortium, Shana Ratner, Leah Frerichs, Kristen Hassmiller Lich, Seth Crockett, Catherine Rohweder, Deeonna E. Farr, Claudia Richardson, Cory Erhard, Evan Beasley, Michelle Hogsed, Ashley Bland

**Affiliations:** 1 Lineberger Comprehensive Cancer Center, University of North Carolina at Chapel Hill, 450 West Drive, Chapel Hill, NC 27599 USA; 2 School of Nursing, University of North Carolina at Chapel Hill, 120 North Medical Drive, Chapel Hill, NC 27599 USA; 3 School of Medicine, University of North Carolina at Chapel Hill, 5034 Old Clinic Building, Chapel Hill, NC 27599 USA; 4 Gillings School of Global Public Health, University of North Carolina at Chapel Hill, 135 Dauer Drive, Chapel Hill, NC 27599 USA

**Keywords:** Cancer screening, Colorectal cancer, Community health centers, Fecal immunochemical test, Patient navigation, Randomized controlled trial, Vulnerable populations

## Abstract

**Background:**

Although colorectal cancer (CRC) screening is effective in reducing CRC mortality, screening rates in vulnerable populations served by community health centers (CHCs) remain below national targets. CHCs in North Carolina are challenged to reach CRC screening targets as they tend to be under-resourced, have limited capacity to implement and sustain population health interventions, and typically operate independently from one another and from regional colonoscopy providers. The Scaling Colorectal Cancer Screening Through Outreach, Referral, and Engagement (SCORE) project is designed to address barriers to CRC screening in partnership with CHCs by implementing a multilevel intervention that includes centralized support infrastructure for mailed fecal immunochemical test (FIT) outreach and patient navigation to follow-up colonoscopy. This paper describes protocols for the SCORE implementation trial.

**Methods:**

We will conduct a type 2 hybrid effectiveness-implementation trial that will assess effectiveness at increasing CRC screening and follow-up rates while also assessing implementation outcomes. The planned trial sample will include 4000 CHC patients who are at average CRC risk and due for screening. Participants will be randomized 1:1 to receive either usual care or a multilevel intervention that includes mailed FIT outreach and patient navigation support to follow-up colonoscopy for those with abnormal FIT. The primary effectiveness outcome is completion of any CRC screening test at six months after randomization. We will also conduct a multilevel assessment of implementation outcomes and determinants.

**Discussion:**

This hybrid effectiveness-implementation trial will evaluate the effectiveness and implementation of an intervention that provides centralized infrastructure for mailed FIT screening and patient navigation for CHCs that operate independently of other healthcare facilities. Findings from this research will enhance understanding of the effectiveness of a centralized approach and factors that determine successful implementation in vulnerable patient populations.

**Trial registration:**

The trial was registered on May 28, 2020, at ClinicalTrials.gov (identifier NCT04406714).

Contributions to the literature

- This study will rigorously evaluate a strategy of providing centralized support for two screening interventions—mailed FIT outreach and patient navigation—for two independent community health care systems. This study will examine the effectiveness and cost-effectiveness of this strategy in this context.
- This study will elucidate implementation barriers and facilitators related to implementing a centralized CRC screening program in a rural region with high CRC incidence and mortality.
- This study will demonstrate application of the Consolidated Framework for Implementation Research for understanding barriers and facilitators to implementing a centrally supported mailed FIT outreach and patient navigation program.

## Background

Despite the availability of effective colorectal cancer (CRC) screening tests and national recommendations for their routine use [1], CRC remains the second leading cause of cancer death in the United States [2]. The CRC burden is particularly heavy in three CRC “hotspots”—regions with elevated CRC mortality rates compared to national averages—including an 11-county region of northeastern North Carolina [3]. National CRC screening rates among adults ages 50–75 years nearly doubled within a 15-year period, up from 34% in 2000 to 62% in 2015 [4]. Disappointingly, screening rates continue to fall short of the national goal of 80% [5].

In North Carolina, as in many parts of the USA, screening rates are particularly low among vulnerable and marginalized populations [6]. The 2018 North Carolina Behavioral Risk Factor Surveillance System survey data revealed that 72% of respondents received one or more recommended CRC screenings within the recommended time interval [7]; however, the CRC screening rate was substantially lower—only 43%—among patients served by the state’s community health centers (CHCs) [8]. These findings highlight substantial disparities in CRC screening for the uninsured, underinsured, and medically underserved populations that rely on CHCs for their healthcare.

The United States Preventive Services Task Force (USPSTF) recommends several tests to screen for CRC, including colonoscopy and fecal blood tests such as fecal immunochemical testing (FIT), for patients ages 50–75 years [9]. Mailed FIT outreach programs can reduce structural barriers to screening by delivering FITs directly to patients’ homes and providing a prepaid envelope to mail the sample to a lab for analysis. Mailed FIT programs have shown promise as an effective means of increasing CRC screening [10–14], including for vulnerable populations [15, 16]. One study demonstrated that a mailed FIT outreach program could increase screening by nearly 30 percentage points compared to usual care among vulnerable patients in a large, safety net system [15]. Mailed FIT may be particularly appealing to populations for whom screening colonoscopy is difficult to access due to transportation, financial, and other barriers [17–19].

Inadequate follow-up after an abnormal (positive) FIT represents a key challenge to effective FIT-based CRC screening. To realize the potential of FIT as a screening modality, it is essential that a positive FIT is followed by a diagnostic (follow-up) colonoscopy. Regrettably, research suggests only 52–58% of patients served by CHCs complete a follow-up colonoscopy after a positive FIT result [20–22]. Further, when colonoscopy follow-up is completed, it is sometimes delayed. This finding is disconcerting because delaying follow-up colonoscopy by 6 months or longer has been associated with higher risk of any CRC and advanced-stage disease [23, 24].

One approach to improving follow-up colonoscopy completion is patient navigation. Although activities vary across settings, patient navigation is a barriers-focused intervention that typically includes identifying and addressing patient, provider, and system-level barriers to appropriate healthcare, as well as providing health education and psychosocial support [25, 26]. Mounting evidence supports the efficacy of patient navigation for improving *screening* colonoscopy completion [10, 27–29], and although it is a promising approach for bolstering *follow-up* colonoscopy completion after a positive fecal blood test [30, 31], additional research is needed in this area, particularly around implementation and cost-effectiveness [10].

CHCs play critical roles in providing primary health care—including CRC screening—for vulnerable populations in North Carolina. Unfortunately, they face numerous challenges to sustaining a robust CRC screening program, including limited resources [32], lack of time [33], high levels of staff turnover [34], and competing priorities [32]. Further, North Carolina’s CHCs are financially and operationally isolated from one another, and rely on multiple electronic health record (EHR) systems. This taxes already limited resources and requires each CHC to develop, implement, and maintain its own population-based CRC screening and follow-up system.

The intervention to be tested in this trial, Scaling Colorectal Cancer Screening Through Outreach, Referral, and Engagement (SCORE), is a multilevel intervention developed as part of the National Cancer Institute-funded consortium The Accelerating Colorectal Cancer Screening and Follow-up through Implementation Science (ACCSIS) Program. The overall aim of ACCSIS is to conduct multi-site, coordinated, transdisciplinary research to evaluate and improve CRC screening processes using implementation science strategies. The SCORE project supports CRC screening at partner CHCs through the development of a centralized, state-level screening outreach support center that will distribute FIT kits to patients and provide navigation for follow-up colonoscopy following a positive FIT result.

The development and testing of the SCORE project has followed the four phases of the Exploration, Preparation, Implementation, and Sustainment (EPIS) framework [35]. A detailed description of the Exploration and Preparation phases will be published separately. The purpose of the current paper is to describe the study design and protocol for the Implementation phase, during which we are conducting a type 2 hybrid effectiveness-implementation trial to test SCORE’s effectiveness at increasing CRC screening and follow-up rates while also assessing its impact on implementation outcomes [36]. A type 2 hybrid design places equal emphasis on examining both effectiveness and implementation. We selected this design because it is aligned with our research aims to assess both the effectiveness and implementation of a centralized support program for delivering mailed FIT outreach and patient navigation to follow-up colonoscopy. Although prior research has established the effectiveness of mailed FIT at improving CRC screening, little is known about the effectiveness of implementing centralized mailed FIT outreach support or about the effectiveness of patient navigation at improving follow-up for positive FIT results in this context. Further, research on implementation outcomes, including costs, will be important for determining the feasibility of taking SCORE to scale statewide and sustaining it over time.

### Aims

As part of the SCORE trial, we will assess the effectiveness, cost-effectiveness, and implementation of a centralized support program for delivering mailed FIT outreach and patient navigation to follow-up colonoscopy. We aim to:

1. Conduct a multi-site, pragmatic randomized controlled trial to assess the impact of the SCORE intervention on CRC screening outcomes in two CHCs in North Carolina (effectiveness aim)
2. Conduct a multilevel assessment of implementation outcomes and determinants (implementation and cost-effectiveness aim)

## Methods/design

### Design

**Fig. 1:**
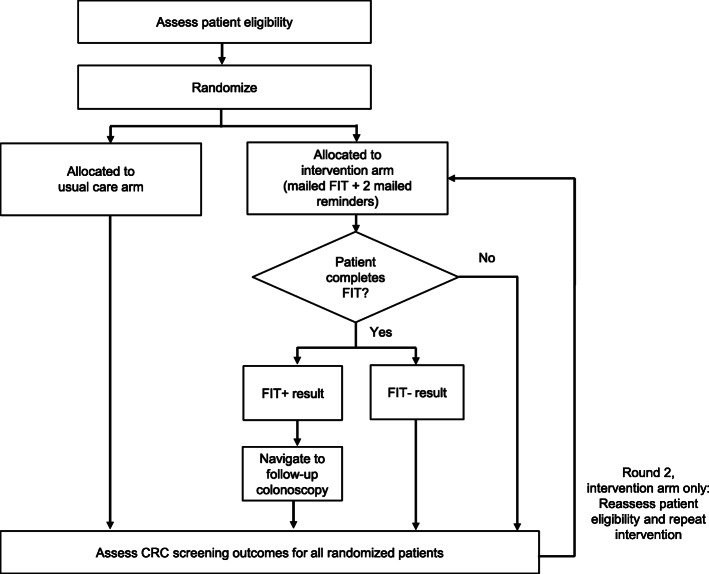
SCORE study flow diagram

SCORE is a type 2 hybrid effectiveness-implementation trial with a two-arm, parallel group, pragmatic randomized controlled trial design. The trial will include 4000 patients at 2 CHCs. To more closely align with procedures that would occur in clinical practice, we will identify eligible patient participants in successive waves, rather than identify the full study cohort at the outset of the trial. For each study wave, we will randomly assign participants 1:1 to usual care or intervention. Participants will remain in their assigned arm for ~ 18 months; this time frame accounts for two rounds of annual FIT plus 6 months to assess CRC screening outcomes after the second round for the intervention arm. The number of waves, number of patients selected for each wave, and timing of the waves will be determined in partnership with participating CHCs and will account for factors such as staffing resources and competing clinical priorities. The study flow is illustrated in Fig. 1. The University of North Carolina at Chapel Hill (UNC Chapel Hill) Institutional Review Board approved this study (protocol # 20-0827).

### Setting

UNC Lineberger Comprehensive Cancer Center (UNC Lineberger) has partnered with two CHCs in North Carolina (“CHC1” and “CHC2”). We will conduct the trial with 12 clinics: 7 clinics of CHC1 and 5 clinics of CHC2. UNC Lineberger will operate as the central location for mailing FITs and providing patient navigation support to study patients served by CHC1 and CHC2. CHC1 serves diverse and vulnerable populations in western North Carolina, including rural Appalachian communities as well as large and growing Hispanic immigrant communities. In 2018, CHC1 served approximately 37,000 patients, of whom 5% were Black/African American, 29% were Hispanic/Latino, 52% were uninsured, and 58% were at or below 100% of the federal poverty level [37]. CHC2 is largely situated in northeastern North Carolina’s 11-county CRC hotspot, with 4 of its 5 clinics located in counties identified in the hotspot [3]. In 2018, CHC2 served nearly 16,000 patients, of whom 56% were Black/African American, 3% were Hispanic/Latino, 13% were uninsured, and 62% were at or below 100% of the federal poverty level [38]. Although the CRC screening rates of 67% (CHC1) [37] and 54% (CHC2) [38] were above the aggregate rate of 43% for CHCs in North Carolina [8], they are well below the national target for 80% in every community [5].

### Study participants

#### Eligibility criteria

We will assess patient eligibility using data from the clinics’ EHR. Eligibility criteria include (1) age 50–75 years, (2) active patient of the clinic (i.e., seen within the past 18 months), (3) at average risk for CRC, (4) no record of CRC diagnosis or total colectomy, (5) no record of comorbidities or screening contraindications, (6) not up to date with CRC screening, and (7) a complete North Carolina mailing address. “Average risk” for CRC is defined as patients with no evidence of prior CRC, colorectal neoplasms, colorectal polyps, colonic adenomas, family history of CRC among first degree relatives (father, mother, sister, brother), or diagnosis of inflammatory bowel disease. Comorbidities and screening contraindications include dementia, hospice care, assisted living, end-stage renal disease, and certain other cancers (glioblastoma, pancreatic cancer, lung cancer, esophageal cancer, liver and bile duct cancer, mesothelioma). Up to date with CRC screening is defined as completing a fecal occult blood test (FOBT) or FIT within the past 12 months, colonoscopy within 10 years, sigmoidoscopy within 5 years, barium enema within 5 years, or computed tomography of the colon (CT colonography) within 10 years. Individuals who do not meet all eligibility criteria at the onset of a given outreach wave will be excluded from participation in that wave but may be reassessed for eligibility for a subsequent wave (e.g., because they became due for screening).

#### Randomization and blinding

Each month during trial enrollment, the CHCs will provide a list of age-eligible patients with a medical encounter in the previous 18 months, generated from the EHR. A study team member will further assess patient eligibility using the aforementioned criteria, then use a randomization sequence generated by the study statistician to randomize participants 1:1 to the intervention and usual care arms, stratified by CHC and primary insurance status (Medicare, Medicaid, commercial/private, self-pay/uninsured), until the sample size for the wave has been reached. We will generate the randomization sequence using permuted block randomization with varying block sizes. Approximately 2000 patients will be randomized to receive the intervention and 2000 will be randomized to usual care. About 1000 patients will be randomized to each arm for each CHC, and we will aim for roughly equal numbers of patients in each insurance stratum, in each arm, at each CHC. Participants will not be blind to intervention assignment; however, intervention arm participants will not be informed that a control arm exists, and control arm participants will not be informed of the research study. For the purposes of outcome assessment from the EHR (to occur at least six months after randomization, see “effectiveness outcomes” below), research staff will be considered blind to study arm even if they had participated in assembly of outreach mailing materials more than 6 months previously.

### Intervention and comparisons

#### Usual care arm

Patients assigned to the usual care arm will receive whatever care they normally would receive. As of the trial onset, usual care at the participating CHCs consisted of visit-based screening, including FIT/FOBT distribution or referral for colonoscopy.

#### Intervention arm

*Mailed FIT outreach*. The mailed FIT outreach consists of a mailed introductory (priming) letter, followed by a FIT kit and up to two reminder letters. The introductory letter recommends CRC screening and alerts the patient that a FIT will arrive in the mail within the next week. The enclosed study information sheet will include a phone number that the patient can call to opt out. Approximately 1 week later, the research team will mail a FIT packet that includes (1) a cover letter, (2) a one-page educational insert about CRC screening and the implications of positive and negative FIT results, (3) an instruction sheet for completing the FIT, (4) a FIT kit (one-sample FIT, tissue paper, biohazard bag, shipping pad), (5) a pre-filled test requisition form, and (6) a pre-paid return envelope to mail the stool sample directly to the lab for processing. UNC research team members will track FIT completion primarily using the lab’s web-based results portal. The research team will mail up to two reminder letters to complete the FIT: the first reminder will be mailed two weeks after the FIT packet, and the second reminder will be mailed a week after the first reminder. Program materials will be provided in both English and Spanish to all patients and mailed in padded envelopes. The envelopes will have a return address label with the CHC’s logo and will not indicate the contents pertain to CRC screening. Because EHR records are based on best available information and may not reflect screenings performed at another facility, SCORE project materials will include a phone number for patients to contact the research team to self-identify as ineligible.

Participants assigned to the intervention arm who remain eligible and due for screening will be sent a second mailed FIT 1 year after the first mailing. Mailing two rounds will allow us to evaluate both annual and biennial screening completion rates [9].

*Patient navigation*. A trained, bilingual English-Spanish patient navigator will reach out to all patients with a positive FIT result after the patients’ provider notifies them of their result and refers them for a follow-up colonoscopy. The patient navigator will follow a navigation protocol adapted from the *Six-Topic Navigation Protocol* developed by the New Hampshire Colorectal Cancer Screening Program [39]. The purpose of patient navigation is to ensure timely follow-up to diagnostic colonoscopy by identifying and addressing barriers to colonoscopy completion, including financial, logistical, and emotional barriers. Centralization is a distinguishing feature of the SCORE navigation implementation approach; the patient navigator has been hired as part of the study team and is located at the outreach support center rather than at the CHCs. The navigator will have real-time access to CHC EHRs and will be able to communicate securely with providers via the EHR. Key components of SCORE patient navigation include (1) timely and supportive telephone contact with patients; (2) addressing patient transportation, financial, and other barriers; (3) ongoing communication and collaboration with staff and providers at the CHCs and endoscopy centers to support patient care; and (4) covering colonoscopy costs for uninsured patients. Although we anticipate most patients will follow a four-call protocol, a smaller proportion likely will require more contacts to resolve barriers and provide needed emotional, logistical, or financial support. Navigation typically will end with a post-colonoscopy follow-up call or handoff to appropriate treatment.

### Outcome measures

#### Effectiveness outcomes

Table 1 provides an overview of effectiveness and cost-effectiveness outcomes, including operational definitions, data source(s), and timing of data collection.

**Table 1:** Effectiveness and cost-effectiveness outcomes for the SCORE project

Outcome	Operational definition	Data source(s)	Timing
CRC screening completion	Proportion of participants who completed CRC screening using any of the screening modalities recommended by the United States Preventive Services Task Force (FIT, FOBT, FIT-DNA, colonoscopy, flexible sigmoidoscopy, flexible sigmoidoscopy with FIT, CT colonography)	EHR chart review	6 months after randomization (round 1) or FIT mail date (round 2)
Mailed FIT completion	Proportion of participants in the intervention (mailed FIT) arm who return a completed FIT	Commercial laboratory’s online portal, REDCap database	60 days, 90 days, and 6 months after randomization (round 1) or FIT mail date (round 2)
Follow-up (diagnostic) colonoscopy completion	Proportion of participants with a positive (abnormal) FIT/FOBT result for whom colonoscopy is deemed to be clinically appropriate by the patient's provider who complete a follow-up (diagnostic) colonoscopy	EHR chart review, REDCap database	6 months after referral for a follow-up colonoscopy
CRCs and advanced adenomas detected	Number of CRCs and advanced adenomas detected among participants who complete a screening or follow-up (diagnostic) colonoscopy	EHR chart review	12 months after referral for a follow-up colonoscopy
Time from CRC diagnosis to the date of evaluation for CRC treatment	Number of days from CRC diagnosis to the date of evaluation for CRC treatment	EHR chart review	Up to 12 months after CRC diagnosis
Referral for cancer treatment	Number of individuals who receive referral for cancer treatment among individuals diagnosed with CRC	EHR chart review	6 months after cancer diagnosis date
Repeat mailed FIT completion	Number of mailed FITs completed (0, 1, or 2) by participants in the intervention (mailed FIT) arm after two rounds of annual FIT outreach	Commercial laboratory’s online portal, REDCap database	Up to 18 months after randomization

The primary effectiveness outcome is CRC screening completion at 6 months after randomization. Screening completion is defined as screening using any of the screening modalities recommended by the USPSTF (FIT, FOBT, FIT-DNA, colonoscopy, flexible sigmoidoscopy, flexible sigmoidoscopy with FIT, CT colonography) [9].

Secondary effectiveness outcomes include (1) proportion of intervention arm participants who return a completed mailed FIT within 60 days, 90 days, and 6 months of randomization; (2) proportion of participants who complete a follow-up (diagnostic) colonoscopy within 6 months after referral for a follow-up colonoscopy; (3) number of CRCs and advanced adenomas detected within 12 months after screening or diagnostic colonoscopy referral; (4) number of days from CRC diagnosis to the date of evaluation for CRC treatment, up to 12 months after CRC diagnosis; (5) number of individuals who receive referral for cancer treatment within 6 months after cancer diagnosis date; and (6) number of mailed FITs completed after two rounds of annual FIT outreach (0, 1, or 2).

#### Implementation outcomes and multilevel implementation determinants

We will use a mixed methods approach to assess implementation outcomes [40] and multilevel implementation determinants [41] from the perspectives of the patients, providers, clinic leaders and frontline staff, and program implementers at the centralized outreach support center. Operational definitions, data sources, and timing of data collection for each measure are presented in Table 2. Of note, the SCORE intervention was specifically designed to use mailed outreach to offload some of the work of CRC screening from clinical CHC staff; however, provider perspectives are an important outcome due to their potential to either encourage or dissuade patient completion of FITs distributed outside of usual care.

**Table 2:** Implementation outcomes and other implementation measures for the SCORE project

Outcome	Operational definition	Data source(s)	Timing
Implementation outcomes			
Reach	Number, proportion, and representativeness of eligible patients who are exposed to mailed FIT and do not opt out Number, proportion, and representativeness of patients with a positive FIT who participate in at least one navigation call	REDCap database	End of trial round 1 and round 2
Acceptability	Perception that the SCORE intervention is agreeable or satisfactory [40]	Clinician stakeholder interview, Patient interview	Clinician stakeholder interview: No earlier than 6 months after start of trial Patient interview: As FIT+ patients are identified; no earlier than 2 weeks after final contact with patient navigator
Appropriateness	Perception that the SCORE intervention is relevant to and compatible with provider’s practice and setting [40]	Provider survey, Clinician stakeholder interview, Patient interview	Provider survey and clinician stakeholder interviews: No earlier than 6 months after start of trial Patient interview: As FIT+ patients are identified; no earlier than 2 weeks after final contact with patient navigator
Cost	The overall cost of the SCORE project; process flow diagrams will serve as a foundation for capturing resource inputs (labor and material costs) for each CRC screening activity	REDCap database, Usual care survey, TAM observation, Patient interview	REDCap database: End of program Usual care survey: No earlier than 6 months after start of trial TAM observation: No earlier than 6 months after start of trial Patient interview: As FIT+ patients are identified; no earlier than 2 weeks after final contact with patient navigator
Cost-effectiveness	Cost per person reached and cost per person screened, compared to usual care	TAM observation, Usual care survey, Administrative data	Up to 36 months after randomization
Feasibility	Perception that the SCORE intervention can be carried out successfully in the CHCs [40]	Clinician stakeholder interview, REDCap database, Patient interview	Clinician stakeholder interview: No earlier than 6 months after start of trial REDCap database: End of program Patient interview: As FIT+ patients are identified; no earlier than 2 weeks after final contact with patient navigator
Fidelity, Adaptations	Fidelity: degree to which the SCORE intervention is delivered as intended [40] Adaptations: adaptations made to the SCORE intervention and reasons for the adaptations	Fidelity: Clinician stakeholder interview, REDCap database, Patient interview Adaptations: Periodic reflection	Clinician stakeholder interview: no earlier than 6 months after start of trial REDCap database: End of program Patient interview: as FIT+ patients are identified; no earlier than 2 weeks after final contact with patient navigator Periodic reflection: ongoing
Other implementation measures			
Multilevel implementation determinants	Barriers and facilitators to SCORE implementation at the level of the • Intervention: evidence strength and quality (CRC screening), relative advantage (centralized mailed FIT and navigation) • Outer setting: patient needs and resources, external policy and incentives • Inner setting: structural characteristics, implementation climate • Individual provider: knowledge and beliefs • Process: engaging/involving team members as champions, reflecting and evaluating	Provider survey, Contextual determinants survey, Clinician stakeholder interview, Periodic reflection	Provider survey, contextual determinants survey, and clinician stakeholder interview: no earlier than 6 months after start of trial Periodic reflection: ongoing

*Implementation outcomes*. The SCORE implementation outcomes were guided by the RE-AIM framework [42] and enhanced by Proctor and colleagues’ conceptual framework for implementation outcomes [40]. We will assess reach, acceptability, appropriateness, cost, cost-effectiveness, feasibility, and fidelity of the mailed FIT outreach and patient navigation components of SCORE (Table 2).

*Reach*. We operationalize reach as the number, proportion, and representativeness of eligible patients who (1) are mailed a FIT kit as part of the outreach intervention and do not contact the study team to opt out and (2) return a FIT kit. We will also assess the number, proportion, and representativeness of patients with a positive FIT who participate in at least one navigation call. To assess reach, we will use data collected in a REDCap (Research Electronic Data Capture) database [43, 44] about patients who (1) were mailed a FIT kit, (2) opted out and reasons for opting out, and (3) for those who had a positive FIT, participation in navigation calls. We will also use patient demographic and clinical characteristics collected in the REDCap database to assess the extent to which the patients reached were representative of the overall eligible population of CHC patients—in other words, the intervention’s impact on equity across factors such as race/ethnicity and sex.

*Acceptability*. We operationalize acceptability as the perception that the SCORE intervention is agreeable or satisfactory [40]. We will conduct interviews to assess patients’ acceptability of SCORE and will code clinician stakeholder interview data for references to acceptability.

*Appropriateness*. We operationalize appropriateness as the perception that the SCORE intervention is relevant to and compatible with the provider’s practice and setting [40]. We will survey providers about the appropriateness of SCORE and will code clinician stakeholder interview data for references to appropriateness. Additionally, we will assess patients’ perceptions of appropriateness as part of the patient interviews.

*Cost and cost-effectiveness*. We will apply micro-costing methods to assess the overall cost of the SCORE project. We will also assess the incremental cost of each additional patient screened in the intervention arm compared to usual care (cost-effectiveness). During the planning phase, we collaborated with the CHCs to develop site-specific process flow diagrams that define and sequence all the activities needed to provide CRC screening at the level of the CHC (as part of usual care procedures) and the level of the centralized SCORE project. These process flow diagrams will serve as a foundation for capturing completion of each activity and the resource inputs (labor and material investments) for each CRC screening and navigation activity. Data collection activities will include (1) periodic episodes of direct observation of mailed FIT outreach activities, such as preparing FIT packets (time and motion [TAM] observation); (2) time logs; (3) self-administered questionnaires and guided interviews with clinic staff to assess labor investment for usual care activity, as well as intervention activity (usual care survey); (4) self-administered questionnaires to assess study staff time spent on implementation activities that happen periodically (e.g., preparing return address labels for mailed materials, inventorying supplies), (5) review of meeting minutes to assess time spent in meetings to plan and monitor implementation, and (6) administrative/financial data to assess materials investments (e.g., supplies and equipment). We will not include time and resources used to develop existing CRC screening tools and patient-facing materials, but we will include such costs as pertinent to adapting these materials for the SCORE intervention and CHC settings. We will conduct in-depth interviews with patients with positive FIT results to understand the amount of time and opportunity costs associated with seeking follow-up colonoscopy and other care.

*Feasibility and fidelity*. Using interviews, we will assess patient and clinician stakeholder perceptions that the SCORE intervention can be carried out successfully in the CHCs (feasibility) and the degree to which the SCORE intervention is delivered as intended (fidelity). We will also use tracking and call log data in the REDCap database to evaluate adherence to a set of prescribed protocols for implementing mailed FIT outreach and patient navigation. As part of our fidelity assessment, we will report the number of items mailed by date as part of the mailed FIT outreach component as well as when and how patients were notified of their FIT result. For the navigation component, we will report adherence to protocols for contacting patients to invite them into the navigation program, as well as dosage (i.e., the total number of navigation contacts and total number of navigation hours) for each patient in the intervention arm with a positive FIT result. We will conduct periodic reflections [45] with CHC staff and program implementers at the outreach support center to regularly reflect on implementation efforts and assess adaptations to the SCORE project. The guided discussions also will provide data on factors affecting implementation outcomes.

*Multilevel implementation determinants*. The Consolidated Framework for Implementation Research (CFIR) [41] guided the development of surveys and interview guides to assess clinic leaders’ and frontline staff experiences with the SCORE project. CFIR has 5 domains—intervention characteristics, outer setting, inner setting, characteristics of individuals, and process—and 39 constructs within those domains.

### Data sources

We will use electronic health records, a REDCap database, surveys, interviews, observation, and periodic reflections to collect data for the SCORE project. Table 3 presents the implementation outcomes and multilevel implementation determinants assessed with each data source.

**Table 3:** Data sources for implementation outcomes and multilevel implementation determinants for the SCORE project

	Data Source							
Construct	REDCap database	Provider survey	Contextual determinants survey	Usual care survey	Clinician stakeholder interview^**1**^	Patient interview	Time and motion (TAM) observation	Periodic reflection
**Implementation outcomes**								
Reach	●							
Acceptability					●	●		
Appropriateness		●			●	●		
Cost	●			●		●	●	
Cost-effectiveness				●			●	
Feasibility	●				●	●		
Fidelity	●				●	●		
Adaptations					●			●
**Other implementation measures**								
Multilevel implementation determinants								
Intervention characteristics		●	●		●			
Outer setting			●		●			
Inner setting			●		●			
Individual provider/practitioner		●	●		●			
Process			●		●			●

^1^Constructs will guide coding of the clinician stakeholder interview data

*REDCap database*. Study personnel at UNC Lineberger will maintain a secure REDCap database that they populate with data from the clinics’ EHRs (see next section on electronic health records). Study personnel also will enter data related to intervention delivery into the REDCap database, including FIT outreach (e.g., mailing dates), FIT results, patient contact with the team, and patient navigation efforts.

*Electronic health records*. Demographic, health history, and screening history data for eligible patients will be obtained via query of each clinic’s EHR and stored in the SCORE project’s REDCap database. Study personnel will assess CRC screening outcomes via manual EHR chart review and enter the data into REDCap. Study personnel will obtain FIT results and dates associated with sample collection and processing from the commercial laboratory’s online portal and will enter the data into the REDCap database.

*Surveys*. We will conduct two brief surveys: a provider survey and a contextual determinants survey. We will administer the surveys at least six months after the SCORE trial launches to allow providers and clinic staff to have some experience with the SCORE intervention.

To develop the provider survey, the study team drew upon items in existing instruments [46–49] as well as created our own items that were grounded in CFIR [41] and Proctor and colleagues’ conceptual framework for implementation outcomes [40]. We then refined items following cognitive interviews with three researchers with expertise in CRC screening, including two clinician-researchers. Items assess providers’ perceptions of the appropriateness and potential barriers and facilitators related to implementing the centralized SCORE intervention as a supplement to their usual care practices. We will administer the online provider survey to all CHC providers who have patients enrolled in the intervention arm of the SCORE trial.

As part of an ACCSIS consortium-wide effort to assess factors that are posited to facilitate or impede implementation of a new CRC screening program (e.g., leadership support and implementation climate), we co-developed a brief contextual determinants survey that we will administer to clinic staff who help implement quality improvement initiatives including SCORE. Survey items were drawn from existing surveys that applied CFIR constructs to assess the multilevel factors that influence CRC screening [41, 46, 47]. We will administer the online survey to clinic leaders and others who have been involved in decisions about implementing, adapting, and sustaining the program (e.g., chief medical officers, SCORE project champions, nurse managers). To estimate the cost of usual care screening, clinic staff also will be asked to complete a survey to help estimate the staff time associated with delivering CRC screening as part of usual care at the clinic (“usual care survey”).

*Interviews*. We will conduct semi-structured interviews with up to 20 clinician stakeholders (e.g., providers, clinic staff) to facilitate interpretation of survey findings and to identify additional factors that may determine successful implementation. We used CFIR to guide the development of the provider interview guide [41]. We will conduct interviews with up to 30 patients who had a positive FIT result to better understand the implementation of the patient navigation component of SCORE and to elucidate patient costs associated with obtaining a follow-up colonoscopy.

*Observation*. We will conduct periodic episodes of direct observation (time and motion study) to assess costs of delivering the mailed FIT outreach component.

*Periodic reflections*. Periodic reflections will be conducted approximately monthly as part of project meetings with CHC staff and program implementers, as well as with individual project staff. Reflections will aid in both understanding factors influencing implementation and capturing clinic-level adaptations to the intervention. The guide for these discussions was adapted from a template developed by Finley and colleagues [45].

### Data analysis plan

We will follow the intention-to-treat principle [50] for our main analyses. Our main statistical test for primary and secondary outcomes will be a Mantel-Haenszel chi-square test, adjusted for recruitment site (CHC). If there are important differences between the control and intervention arms across baseline variables known to be associated with the outcome being tested, we will also then use multiple logistic regression, adjusting for the additional baseline variables. One-sided tests will be used for the primary and secondary outcomes because we expect the active intervention will lead to more favorable results (increased screening completion) compared to usual care.

Cost data for the intervention and usual care alternatives will be aggregated across data sources, by activity, guided by our process flow diagrams. Costs will be categorized as fixed or variable and personnel or non-personnel (supplies/equipment) for micro-costing summaries. Calculations will report the total cost of the SCORE intervention, as well as cost per person reached and cost per person screened, compared to usual care. Sensitivity analyses will separately assess the value of reminder mailings and cost differentials by key groups (e.g., insurance type and CHC site).

Interviews and periodic reflections will be audio-recorded and transcribed. We will use qualitative software (e.g., NVivo) to organize and manage the data. The interview guide will serve as the foundation for developing an a priori codebook based on CFIR, with emergent codes added to the codebook as needed after reviewing and writing memos on the transcripts. Two team members will read and briefly summarize the interviews/reflections, will code separately, and meet to discuss codes and come to agreement on interpretation. Conflicting interpretations will be resolved through discussion with the larger team, as needed. We will use memo writing [51] and data displays (e.g., matrices) [52] throughout the analysis to inform interpretation and help make sense of the findings.

### Sample size and power

The study statistician (XT) performed formal power calculations for the primary study outcome comparing CRC screening completion for the usual care arm vs. the intervention arm for each stratum defined by patient insurance status (4 strata: Medicare, Medicaid, commercial/private, and self-pay/other). These calculations assume equal numbers of participants at each site, in each arm, and in each stratum. Based on previous research [53], we aimed to power our study such that we would have 80% power to detect an 8% difference in the proportion screened between the intervention and usual care arms. We used SAS PROC Power (SAS Institute, Cary, NC) to determine that we will need 492 participants per arm, per strata (3936 participants total, which can be rounded to 4000) to have at least 80% power to detect an 8 percentage point difference between study arms (assuming a 17% screening rate for the usual care arm) at a 1-sided alpha level of 0.0125 (Bonferroni adjustment for multiple tests to ensure overall type 1 error is under 0.05).

## Discussion

We have presented a protocol describing planned pragmatic research to assess the effectiveness and implementation outcomes of a centralized intervention to improve CRC screening among vulnerable patient populations served by CHCs. Although previous research demonstrates that mailed FIT outreach and follow up is effective and can be implemented in large, integrated health care systems [54], the planned study will examine an implementation approach that aims to increase screening by supporting smaller, non-integrated CHCs with centralized outreach support.

In carrying out this research, we foresee encountering challenges common to pragmatic implementation studies, particularly when conducted in low-resource contexts. Some of the anticipated potential challenges include staffing turnover within CHCs, regulatory barriers to data sharing, changes in EHR systems, changing priorities and resources at the CHC level, and the emergence of new technologies, policies, and/or clinical guidelines relevant to CRC screening. Nevertheless, we also expect that the need to adapt our interventions and strategies in response to dynamic contextual challenges will also provide opportunities to study these adaptations rigorously, and this in turn will yield greater understanding of these complex implementation issues.

The proposed study will take place as part of a larger consortium in collaboration with scientific partners from the National Cancer Institute, RTI International, and other funded research sites across the United States. As such, it will contribute to a “bigger picture” understanding of how to improve implementation of interventions to improve CRC screening in diverse, vulnerable populations in a variety of contexts. Thus, we anticipate that the individual and collective findings will accelerate progress in the fields of both cancer prevention and control and implementation science. We intend to disseminate these findings through publications in peer-reviewed journals and presentations at meetings with scientific and key stakeholder audiences.

## Data Availability

Not applicable

## Abbreviations

ACCSIS: Accelerating Colorectal Cancer Screening and Follow-up through Implementation Science
CFIR: Consolidated Framework for Implementation Research
CHC: Community health center
CRC: Colorectal cancer
CT: Computed tomography
EHR: Electronic health record
FIT: Fecal immunochemical test
FOBT: Fecal occult blood test
REDCap: Research Electronic Data Capture
SCORE: Scaling Colorectal Cancer Screening Through Outreach, Referral, and Engagement
TAM: Time and motion
UNC: University of North Carolina
USPSTF: United States Preventive Services Task Force
